# Redox regulation in aging muscles: exercise as a key modulator to combat sarcopenia and frailty

**DOI:** 10.3389/fcell.2026.1772623

**Published:** 2026-03-17

**Authors:** Hua Guo, Xueqin Jiang, Wang Zhiming, Yang Gui, Zhanguo Su

**Affiliations:** 1 Department of General Practice, The Affliated Wuxi People’s Hospital of Nanjing Medical University, Wuxi Medical Center, Nanjing Medical University, Wuxi People’s Hospital, Wuxi, Jiangsu, China; 2 Department of Geriatrics, Shanghai health and medical center, Wuxi, Jiangsu, China; 3 Faculty of Physical Education, Huainan Normal University, Huainan, Anhui, China; 4 Physical Institute of Nanchang Institute of Technology, Nanchang, China

**Keywords:** antioxidant defense, exercise, mitochondrial biogenesis, muscle aging, oxidative stress

## Abstract

**Background and Context:**

Aging is characterized by progressive decline in skeletal muscle function, which can lead to sarcopenia (loss of muscle mass and strength) and frailty (increased vulnerability to stressors), with oxidative stress—arising from an imbalance between reactive oxygen species (ROS) production and antioxidant defenses—playing a central role. This narrative review synthesizes evidence on how exercise modulates redox homeostasis to mitigate these conditions in older adults.

**Objectives:**

To explore the sources and consequences of oxidative stress in aging muscle, examine exercise’s role in restoring redox balance, evaluate its impact on sarcopenia and frailty, and identify relevant biomarkers and future research directions. We achieve this by exploring key sources through representative studies, examining molecular mechanisms via pathway analyses, evaluating intervention effects using RCTs and meta-analyses, and identifying biomarkers and gaps through critical synthesis.

**Methods:**

This narrative review involved a comprehensive literature search in databases such as PubMed, Web of Science, and Scopus, focusing on studies from 2000 to 2025 on oxidative stress, exercise, sarcopenia, and frailty in adults aged 60+. Inclusion criteria prioritized peer-reviewed articles, meta-analyses, and RCTs; exclusion applied to non-English or irrelevant studies. Over 100 articles were selected qualitatively for synthesis.

**Key Findings:**

Aerobic and resistance exercises reduce oxidant markers (e.g., MDA decreased by 10%–20% in meta-analyses) and enhance antioxidants (e.g., SOD increased by 15%–30%), upregulating pathways like Nrf2, AMPK, and PGC-1α. Multicomponent programs improve muscle strength (e.g., 20%–40% gains in RCTs) and frailty scores (e.g., reductions in Fried Frailty Phenotype by 1–2 points). However, heterogeneous responses exist, with some studies showing neutral effects on certain markers.

**Conclusion:**

Exercise emerges as a non-pharmacological intervention to attenuate oxidative stress-driven muscle aging, promoting healthy aging. Future studies should focus on personalized regimens and long-term biomarkers for clinical translation.

## 1 Introduction

### 1.1 Research background

A complex biological process, aging is marked by a slow build-up of cellular and molecular changes that show up as a reduction in physiological function and a higher risk of illness (Militello et al., 2024). This process is not merely a function of time but involves active metabolic and physicochemical changes within cells (Militello et al., 2024). Among the significant health challenges associated with advancing age are sarcopenia and frailty, which affect millions globally as the population ages, with projections estimating over 2 billion people aged 60+ by 2050 (Militello et al., 2024; Simioni et al., 2018). Sarcopenia is a geriatric syndrome defined by the age-associated reduction in skeletal muscle mass, strength, and functionality (Maldonado et al., 2023). It is recognized as a unique clinical entity that has significant effects on older adults’ independence and general health (Maldonado et al., 2023). Conversely, frailty is a state of increased susceptibility to stressors brought on by a reduction in physiological reserves in a number of organ systems (Anwar et al., 2023). It is a multidimensional syndrome that often precedes disability and elevates the risk of adverse health outcomes (Álvarez-Satta et al., 2020).

### 1.2 Current issues

Oxidative stress is a fundamental factor associated with the pathophysiology of aging and its associated disorders (Dai et al., 2023). The fundamental cause of oxidative stress is an imbalance between ROS production and the body’s antioxidant defense mechanisms (Ye et al., 2021). This imbalance can damage cells and cause age-related diseases (Maldonado et al., 2023). Key issues include the rising prevalence of sarcopenia (affecting 10%–50% of older adults) and frailty (up to 25% in community-dwelling elderly), leading to increased healthcare costs, falls, and mortality (Gomes et al., 2017; Chen et al., 2020). Current interventions are limited, with pharmacological options showing mixed efficacy and side effects.

### 1.3 Mechanisms and effects

Aging mechanisms involve mitochondrial dysfunction, proteostasis loss, and cellular senescence, exacerbated by oxidative stress (Kadoguchi et al., 2020; Maldonado et al., 2023). Phenotypes include muscle atrophy (Type II fibers more affected), reduced strength, and impaired regeneration (Gomes et al., 2017; Shengchen et al., 2021). Exercise, as a modifiable lifestyle factor, may affect oxidative stress levels, subsequently influencing muscle aging processes and the onset of frailty in older adults (Ji, 2001). Aerobic exercise enhances mitochondrial efficiency, while resistance training promotes protein synthesis; combined, they reduce ROS and inflammation (Atashak and Azizbeigi, 2017; Ye et al., 2021). Effects include improved muscle resilience (e.g., 15%–30% strength gains) and reduced frailty risk (Álvarez-Satt et al., 2020; Prommaban et al., 2024).

### 1.4 Objectives and significance

This report seeks to consolidate the existing scientific knowledge regarding the complex interplay among exercise, oxidative stress, sarcopenia, and frailty in the elderly population. Specific objectives include: investigating how different exercise types modulate oxidative stress; exploring mechanisms linking oxidative stress to sarcopenia; analyzing relationships with frailty; evaluating exercise intervention effects; and discussing biomarkers. We achieve these by exploring key sources and consequences through representative studies and meta-analyses, examining exercise’s modulatory role via molecular pathway analyses, evaluating impacts on sarcopenia and frailty using RCTs and observational data, and identifying biomarkers and research directions through critical gaps synthesis. The significance lies in providing evidence-based guidance for exercise prescriptions to promote healthy aging, reduce healthcare burdens, and enhance quality of life in an aging society. Based on prior evidence, exercise, a modifiable lifestyle factor, may affect oxidative stress levels, subsequently influencing muscle aging processes and the onset of frailty in older adults (Ji, 2001). The global population of older adults is steadily increasing, underscoring the importance of identifying strategies to maintain wellbeing and prevent age-related decline (Militello et al., 2024). This report seeks to consolidate the existing scientific knowledge regarding the complex interplay among exercise, oxidative stress, sarcopenia, and frailty in the elderly population. This study will investigate how different types and intensities of exercise modulate oxidative stress, explore the mechanisms by which oxidative stress contributes to sarcopenia, analyze the relationship between these factors and frailty, evaluate the effects of exercise interventions, and discuss relevant biomarkers.

## 2 Methods

This narrative review synthesizes literature on redox regulation, exercise, sarcopenia, and frailty in aging muscles. The search was conducted in January 2026 across PubMed, Web of Science, Scopus, and Google Scholar. Specific search strategies included:

- PubMed: ((“oxidative stress” [MeSH Terms] AND “aging” [MeSH Terms] AND “muscle, skeletal” [MeSH Terms]) OR (“exercise” [MeSH Terms] AND “sarcopenia” [MeSH Terms]) OR (“frailty” [MeSH Terms] AND “reactive oxygen species” [MeSH Terms]) OR (“biomarkers” [MeSH Terms] AND “exercise” [MeSH Terms] AND “aged” [MeSH Terms])) AND (“2000/01/01” [Date - Publication]: “2025/12/31” [Date - Publication]); English language; peer-reviewed.
- Web of Science: TS=(“oxidative stress” AND “aging muscle”) OR TS=(“exercise” AND “sarcopenia”) OR TS=(“frailty” AND “ROS”) OR TS=(“biomarkers” AND “exercise elderly”), refined by year (2000–2025), document type (article/review), language (English).
- Scopus: TITLE-ABS-KEY (“oxidative stress” AND “aging muscle” OR “exercise” AND “sarcopenia” OR “frailty” AND “ROS” OR “biomarkers” AND “exercise elderly”) AND PUBYEAR >1999 AND PUBYEAR <2026 AND [LIMIT-TO (LANGUAGE, “English”)] AND (LIMIT-TO (DOCTYPE, “ar”) OR LIMIT-TO (DOCTYPE, “re”)].
- Google Scholar: Advanced search with keywords “oxidative stress aging muscle exercise sarcopenia frailty biomarkers”, years 2000–2025, excluding patents/citations.

Initial hits: PubMed (n = 512), Web of Science (n = 428), Scopus (n = 365), Google Scholar (n = 1,200; top 200 reviewed). After removing duplicates (n = 345), 2,160 records were screened by title/abstract for relevance to oxidative stress, exercise interventions, sarcopenia/frailty in older adults (≥60 years). Full-text assessment (n = 320) led to exclusions for irrelevance (n = 85), non-human focus without translation (n = 72), low quality (n = 59; e.g., unclear methods). 104 articles were included, prioritizing high-impact RCTs, meta-analyses, and reviews. A PRISMA-inspired flow (adapted for narrative review) is described here; a diagram will be provided as Supplementary Figure S1. Articles were qualitatively synthesized thematically (e.g., mechanisms, interventions), with no formal risk of bias assessment but prioritization of stronger designs (RCTs > observational). The 104 included articles are detailed in Supplementary Table S1 (e.g., author, year, design, population, key findings). This approach minimizes selection bias while allowing broad synthesis; limitations are discussed later.

## 3 Oxidative stress in the elderly: sources, mechanisms, and consequences

With advancing age, several factors contribute to raise in oxidative stress within the body. A primary source of elevated ROS levels in older adults is mitochondrial dysfunction (Zhang et al., 2023). As mitochondria age, their efficiency in producing adenosine triphosphate (ATP) diminishes, and they become more prone to generating ROS as byproducts (Chistiakov et al., 2014). The overall oxidative burden is significantly influenced by this age-related decline in mitochondrial function (Maldonado et al., 2023). Furthermore, the body’s antioxidant defense mechanisms also decline with age (Kozakiewicz et al., 2019). The activity of key antioxidant enzymes, like catalase, glutathione peroxidase, and SOD, is often reduced in older individuals, impairing their ability to neutralize ROS effectively (Militello et al., 2024; Sullivan-Gunn and Lewandowski, 2013; Mao et al., 2019). In In addition to mitochondrial sources, xanthine oxidase and NADPH oxidase increase ROS production in aging organisms (Gomes et al., 2017; Shin et al., 2008; Vida et al., 2011). Over time, molecular damage that affects DNA, proteins, and lipids accumulates as a result of the combined effects of increased ROS generation and decreased antioxidant defense (Chen et al., 2022a).

At the molecular and cellular levels, this increased oxidative stress manifests in several damaging ways. Lipid peroxidation, a process where ROS damage lipids, can alter the fluidity and function of cell membranes (Ni et al., 2023). This can compromise cellular integrity and disrupt normal physiological processes (Ammendolia et al., 2021). Proteins are vulnerable to oxidative damage, which encompasses carbonylation and glycation (Ershler, 2007). These modifications can impair protein structure and activity, leading to disruptions in various cellular functions (Davies, 2016). Furthermore, ROS can cause DNA damage, contributing to genomic instability and cellular senescence (Benkafadar et al., 2019). This accumulation of damage to essential cellular components contributes to the aging phenotype (Chen et al., 2022a).

**FIGURE 1:**
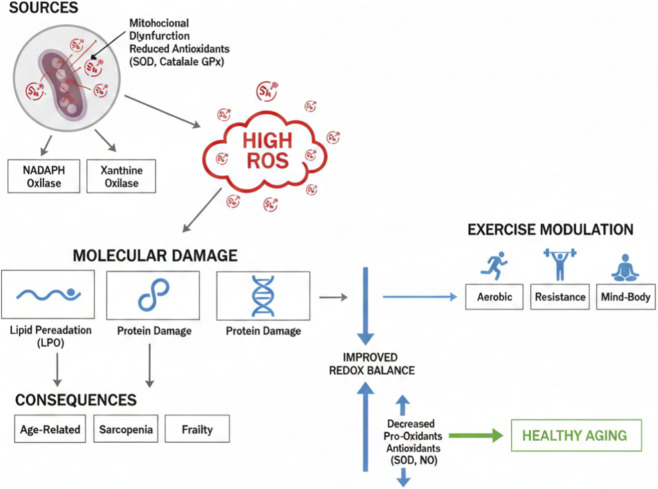
Oxidative Stress in Aging: Sources, Consequences, and Exercise Modulation. This integrated diagram illustrates primary sources (mitochondrial dysfunction, reduced antioxidants like SOD/catalase/GPx, NADPH/xanthine oxidases) leading to high ROS, molecular damage (lipid peroxidation, protein/DNA damage), and outcomes (age-related diseases, sarcopenia, frailty). Exercise modalities (aerobic/resistance/combined/mind-body) reduce pro-oxidants (e.g., MDA, LPO) and enhance antioxidants (e.g., SOD, NO), improving redox balance for healthy aging.

Increased oxidative stress in older adults has extensive implications. This is associated with the emergence and progression of multiple age-related diseases, including cardiovascular disease, cancer, and neurodegenerative disorders (Junior et al., 2019; Hassan et al., 2022; Huang et al., 2024). Oxidative stress significantly contributes to muscle aging and sarcopenia through various mechanisms, which will be elaborated upon later in this report. Additionally, increased oxidative stress is linked with the onset of frailty, which is defined by diminished physiological reserve and heightened susceptibility to negative health outcomes (Soysal et al., 2017). The systemic decline associated with frailty is often accompanied by increased levels of oxidative damage (Anwar et al., 2023) (Figure 1).

### 3.1 Exercise effects on oxidative stress: acute vs. chronic adaptations

The effects of exercise on oxidative stress in older adults are complex, differing between acute and chronic responses. The acute and chronic effects of exercise on oxidative stress in older adults are complex. During intense or prolonged exercise, there is a temporary increase in the production of ROS due to heightened metabolic activity and oxygen consumption (Bouzid et al., 2015). This phenomenon is often referred to as “exercise-induced oxidative stress” (Militello et al., 2024). The primary sources of ROS during physical exertion are the mitochondria, where increased aerobic metabolism leads to the generation of reactive intermediates, including superoxide and hydrogen peroxide (Powers and Jackson, 2008). Additionally, enzymes like NADPH oxidase and xanthine oxidase, found in different regions of the muscle cell, contribute to the production of ROS during physical activity (Powers and Jackson, 2008; Henríquez-Olguin et al., 2019; Gomez-Cabrera et al., 2010). Studies have observed increases in markers of oxidative stress, like MDA and lipid peroxidation products, following acute bouts of exercise (Ni et al., 2023; Spirlandeli et al., 2014). Furthermore, physiological changes occurring during exercise, such as blood flow redistribution leading to hypoxia and subsequent reperfusion, can also contribute to lipid peroxidation (Ni et al., 2023).

In contrast, regular exercise has been shown to induce chronic adaptations in the body’s antioxidant systems (Militello et al., 2024). Regular exercise boosts catalase, SOD, and glutathione peroxidase activity (Xu et al., 2022; Shields et al., 2018; Wibawa et al., 2024). Active elderly individuals typically demonstrate reduced basal levels of oxidative stress in comparison to their sedentary peers of equivalent age (Militello et al., 2024). This suggests that maintaining an active lifestyle can help to reduce the overall oxidative burden in older adults (Grossini et al., 2024). The concept of hormesis may also play a role, where the mild, repeated stress imposed by exercise triggers adaptive responses within cells, ultimately enhancing their resilience to more severe stressors (Militello et al., 2024). Regular exercise can also improve mitochondrial function, potentially leading to a reduction in ROS production over the long term (Wang F. et al., 2021).

### 3.2 Factors influencing the relationship

The complex relationship between exercise and oxidative stress in older adults is affected by many factors. These include aerobic, resistance, or a combination of exercise, intensity (low, moderate, or high), duration and frequency of sessions, baseline antioxidant capacity, training status, and pre-existing health conditions (Militello et al., 2024; Wang X. et al., 2021; Lima et al., 2022; Thirupathi et al., 2021). Thus, the impact of exercise on oxidative stress depends on how these factors interact (Militello et al., 2024; Wang X. et al., 2021; Thirupathi et al., 2021; El Assar et al., 2022). This sets the stage for understanding key molecular mechanisms, as discussed below.

### 3.3 Key molecular mechanisms Nrf2, AMPK, and PGC-1α

Central to exercise’s modulation of oxidative stress are molecular cascades such as Nuclear factor erythroid 2-related factor 2 (Nrf2) activation, AMP-activated protein kinase (AMPK) signaling, and Peroxisome proliferator-activated receptor gamma coactivator 1-alpha (PGC-1α) regulation. Nrf2 functions as a master transcription factor that regulates over 250 genes involved in antioxidant defense, detoxification, and cellular protection (Zhang et al., 2024). In aging skeletal muscle, Nrf2 activity declines, leading to heightened oxidative stress, impaired mitochondrial biogenesis, and accelerated sarcopenia (Zhang et al., 2024; Huang et al., 2019). Its regulatory pathway involves dissociation from Keap1 under oxidative stress or exercise stimuli, translocation to the nucleus, and binding to antioxidant response elements (AREs) to upregulate enzymes like SOD, GPx, and heme oxygenase-1 (HO-1) (Zhang et al., 2024). Exercise, particularly endurance training, activates Nrf2 by increasing ROS as a signaling molecule, enhancing stem cell proliferation/differentiation, restoring mitochondrial dynamics, and reducing frailty (Zhang et al., 2024; Huang et al., 2019; Lu et al., 2021). In Nrf2-deficient models, exercise capacity diminishes, emphasizing its role in muscle resilience (Zhang et al., 2024).

AMPK acts as an energy sensor, activated by increased AMP/ATP ratios during exercise or metabolic stress, phosphorylating targets to promote catabolism and inhibit anabolism (Fan and Evans, 2017). In aging muscle, AMPK signaling wanes, contributing to energy deficits, inflammation, and sarcopenia (Neto et al., 2023; Angulo et al., 2020). Its pathway intersects with PGC-1α and Nrf2; AMPK phosphorylates PGC-1α to enhance mitochondrial biogenesis and activates Nrf2 for antioxidant responses (El Assar et al., 2022; Fan and Evans, 2017). Exercise restores AMPK in older adults, reducing oxidative damage, improving insulin sensitivity, and countering frailty, with resistance training showing pronounced effects on muscle proteostasis (Militello et al., 2024; Fan and Evans, 2017).

PGC-1α is a coactivator that orchestrates mitochondrial biogenesis, fatty acid oxidation, and antioxidant defenses by interacting with transcription factors like ERRα and NRF-1/2 (Neto et al., 2023; Song et al., 2024). In sarcopenia, PGC-1α downregulation leads to mitochondrial dysfunction, ROS overload, and muscle atrophy (Song et al., 2024; Halling et al., 2019). Its regulatory pathway is modulated by AMPK (phosphorylation) and SIRT1 (deacetylation), with exercise inducing PGC-1α expression to neutralize ROS, promote fiber remodeling, and interact with Nrf2 for synergistic effects (Song et al., 2024; Halling et al., 2019). In older models, PGC-1α modulation via aerobic exercise mitigates oxidative stress and frailty, though responses vary by intensity and individual factors (Song et al., 2024; Gureev et al., 2019). These pathways collectively foster muscle resilience, though heterogeneous responses warrant personalized approaches (Gureev et al., 2019).

## 4 Impact of exercise type and intensity on oxidative stress modulation

### 4.1 Aerobic exercise

Aerobic exercise has been thoroughly examined regarding its impact on oxidative stress in older adults (Bouzid et al., 2014a). Meta-analyses indicate that regular aerobic exercise positively affects oxidative stress levels by decreasing blood oxidant markers and increasing antioxidant marker levels (Ye et al., 2021; Zhao et al., 2023). Specifically, studies have shown that aerobic exercise can significantly reduce levels of MDA and LPO, another indicator of oxidative damage to lipids (Ye et al., 2021; Baghaiee et al., 2016; Ghoraba et al., 2022). Additionally, aerobic exercise has been found to increase the levels of NO and SOD (Ye et al., 2021; Baghaiee et al., 2016; Ozkol et al., 2012). Generally speaking, low to moderate intensity—between 40% and 80% of the person’s stored maximum heart rate—is advised to produce these positive effects (Ye et al., 2021). Some studies have reported conflicting findings, indicating no effect of running exercise on certain oxidative stress markers, which suggests that the specific type of aerobic exercise may influence the outcome (Ye et al., 2021).

### 4.2 Resistance training

Resistance training has been demonstrated to influence oxidative stress in older adults (Vezzoli et al., 2019). Research indicates that regular resistance training can strengthen the body’s antioxidant system (Di Lorito et al., 2021). Furthermore, low-intensity resistance training (LIRT) combined with aerobic exercise may be particularly effective in dampening cellular lipid peroxidation in the elderly (Ni et al., 2023). There is evidence that resistance training can lower levels of homocysteine and oxidative stress that are induced by exercise. This suggests that resistance training may have the potential to alleviate the acute oxidative stress response that is associated with physical activity (Atashak and Azizbeigi, 2017).

### 4.3 Combined training

Combined training, which incorporates both resistance and aerobic exercises, appears to be a particularly beneficial strategy for boosting redox balance in older adults (Bachi et al., 2019). A study with elderly men showed that 8 weeks of combined training resulted in declined pro-oxidant markers, including hs-CRP, MDA, and TNF-α, alongside an increase in the antioxidant enzyme SOD (Atashak and Azizbeigi, 2017). This suggests that a comprehensive exercise program addressing both cardiovascular fitness and muscle strength can have a significant positive impact on oxidative status (Cheragh Birjandi et al., 2023).

### 4.4 Mind-body exercises

**FIGURE 2:**
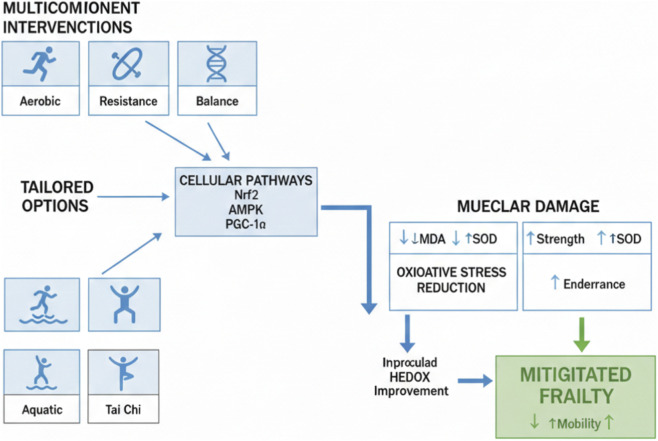
Exercise Interventions for Elderly Health: Pathways and Outcomes. This diagram depicts multicomponent interventions (aerobic/resistance/balance/flexibility) activating Nrf2/AMPK/PGC-1α to reduce oxidative stress (e.g., ↓MDA, ↑SOD), improve muscle health (↑strength/endurance), and mitigate frailty (↓vulnerability, ↑mobility). Tailored for frail elderly, with low-intensity options like aquatic/Tai Chi.

Tai Chi, Yoga, and other exercise methods may reduce oxidative stress in individuals, particularly those with underlying health conditions (Lu et al., 2021; Rosado-Pérez et al., 2021; Gupta et al., 2020). Baduanjin, a traditional Chinese exercise, has been found to reduce pro-oxidative markers like 8-iso-PGF2α and MDA and increase the antioxidant enzyme SOD in older adults (Ye et al., 2024) (Figure 2; Tables 1, 2).

**TABLE 1:** Impact of various exercise modalities on oxidative stress, muscle health, and frailty in older adults.

Exercise type	Intensity	Duration/Frequency	Study population	Study design/Ref	Oxidative stress outcomes	Muscle health outcomes	Frailty outcomes
Aerobic	Low to Moderate (40%–80% max HR)	Regular (e.g., 4–12 weeks, 3–5x/week)	Older adults (≥60 years)	Meta-analysis/RCT (Dai et al., 2023; Xu et al., 2022)	Reduced MDA (10%–20%), LPO; Increased NO, SOD (15%–30%), TAC, Vitamin E	Improved function (e.g., endurance, SPPB ↑20%)	Not specified; indirect via reduced vulnerability
Resistance	Low to Moderate	Regular (e.g., 8–12 weeks, 2–3x/week)	Older adults (≥60 years)	RCT (Wang et al., 2021a; Wang et al., 2024)	Strengthened antioxidant system; Reduced acute stress (e.g., ↓homocysteine)	Increased strength/mass (e.g., MVC ↑30%–40%)	Reduced frailty scores (FFP ↓1 point)
Combined (aerobic + resistance)	Moderate	8–14 weeks, 3x/week	Overweight/inactive elderly men (≥65 years)	RCT (Lima et al., 2022; El Assar et al., 2022)	Reduced TNF-α, hs-CRP, MDA; Increased SOD	Improved endurance/stability (e.g., TUG ↓15%)	Improved status (EFS ↑10%–20%)
Multicomponent (aerobic + resistance + balance + flexibility)	Moderate to High	14–40 weeks, 2–5x/week	Frail/pre-frail older Adults (≥70 years)	Meta-analysis (Hyatt et al., 2019; Chen et al., 2022a)	Reduced inflammatory/oxidative markers (e.g., ↓IL-6)	Improved strength (HGS ↑25%), mobility (gait speed ↑20%)	Improved FFP/EFS (↓1–2 points), emotional wellbeing
Tai chi/yoga	Low	Long-term (e.g., 12–24 weeks, 3x/week)	Unhealthy older adults (≥60 years)	RCT/Meta (Zhang et al., 2024; Huang et al., 2019; Lu et al., 2021)	Reduced MDA; Increased SOD	Improved balance/flexibility	Reduced frailty risk
Baduanjin	Low to Moderate	24 weeks, 3x/week	Older adults with cognitive frailty (≥65 years)	RCT (Fan and Evans, 2017)	Reduced MDA, 8-iso-PGF2α; Increased SOD	Not specified	Improved cognitive frailty (MoCA ↑15%, EFS ↓20%)
Aquatic (low-intensity aerobic)	Low	12–24 weeks, 2–3x/week	Elderly with depression/mobility limits (≥65 years)	RCT (Wang et al., 2021b; Hajam et al., 2022)	Decreased oxidative stress/anxiety	Improved functional anatomy	Not specified

**TABLE 2:** Evidence strength summary for exercise effects.

Exercise type	Study design	Primary conclusions	Evidence strength (high/Moderate/Low)	Key refs
Aerobic	Meta-analyses/RCTs	Reduces oxidants, enhances antioxidants; variable on specific markers	High (consistent meta-data)	Dai et al. (2023), Xu et al. (2022)
Resistance	RCTs	Boosts antioxidants, improves muscle; limited frailty data	Moderate (fewer meta-analyses)	Wang et al. (2021a), Wang et al. (2024)
Combined	RCTs	Broad redox/muscle/frailty benefits	High (multiple RCTs)	Lima et al. (2022), El Assar et al. (2022), Hyatt et al. (2019)
Mind-Body (Tai Chi/Baduanjin)	RCTs/Meta	Reduces stress markers, improves frailty	Moderate (emerging evidence)	Zhang et al. (2024), Huang et al. (2019), Lu et al. (2021), Fan and Evans (2017)
Multicomponent	Meta-analyses	Comprehensive improvements	High (strong consistency)	Hyatt et al. (2019), Chen et al. (2022a)

### 4.5 Heterogeneity in exercise responses and discrepancies across studies

Although exercise generally modulates oxidative stress positively, responses are heterogeneous, with some studies reporting neutral or contradictory effects (e.g., no change in MDA post-aerobic training) (Ye et al., 2021). This variability stems from differences in study subjects, intervention methods, prescriptions, and outcome interpretations (Buford et al., 2014; Sparks, 2017). For instance, participant factors like baseline fitness, age, sex, hormonal status, comorbidities (e.g., frailty vs. healthy), and genetics influence outcomes; frail individuals may exhibit faster energetic decline and lower mitochondrial capacity, leading to neutral effects at higher intensities (Buford et al., 2014; Sparks, 2017; Andreux et al., 2018). Exercise intensity explains efficacy differences within modalities—low-moderate (40%–60% max HR) often reduces oxidants in untrained elderly, while high-intensity (>80%) may exacerbate acute stress in those with poor perfusion or chronic diseases due to excessive ROS production (Buford et al., 2014; Sparks, 2017). Discrepancies (e.g., effective in one study, none in another) arise from physiological foundations: e.g., insulin resistance or inflammation blunts adaptations in obese participants, while trained individuals show amplified benefits (Sparks, 2017). Suitable populations include pre-frail for low-intensity aerobic (e.g., walking) to build reserves, and healthy elderly for combined training; contraindications involve high-intensity for those with cardiovascular risks or severe frailty, risking overstress (Andreux et al., 2018). Addressing these allows extraction of practical value from the 104 studies, emphasizing personalized prescriptions.

## 5 Oxidative stress in muscle aging and sarcopenia

Oxidative stress is defined as an imbalance between the production of reactive oxygen species (ROS) and the body’s ability to detoxify them via antioxidants, potentially leading to cellular damage (Sies, 2015). Skeletal muscle aging refers to the gradual deterioration of muscle structure and function with advancing age, including atrophy and reduced regenerative capacity (Cruz-Jentoft and Sayer, 2019). Sarcopenia is a progressive syndrome characterized by the loss of skeletal muscle mass, strength, and performance, often diagnosed via criteria like low appendicular lean mass and grip strength (Chen et al., 2020; Cruz-Jentoft and Sayer, 2019). While skeletal muscle aging encompasses a broad range of age-related changes, sarcopenia represents a more severe manifestation where these changes reach a clinical threshold, significantly impacting physical performance and quality of life (Dao et al., 2020; Lim and Frontera, 2023). Their interrelationships form a vicious cycle: oxidative stress exacerbates muscle aging by damaging mitochondria and proteins, which in turn promotes sarcopenia phenotypes; conversely, sarcopenia-associated mitochondrial dysfunction and reduced physical activity leading to decreased mitochondrial function result in further increases in ROS, amplifying stress and frailty risk (Maldonado et al., 2023; Sies, 2015; Cruz-Jentoft and Sayer, 2019; Wang et al., 2024). This logical progression—oxidative stress → molecular/cellular damage → muscle atrophy and dysfunction—underpins the section’s framework, avoiding reversed causality. Chronic oxidative stress induced by aging drives sarcopenia but is not an inducing cause of general skeletal muscle aging, whereas increased oxidative stress from exogenous stimuli (e.g., environmental factors) can promote broader skeletal muscle aging under differing preconditions (Chen et al., 2022a; Wang et al., 2024). Oxidative stress is closely associated with the essential mechanisms of muscle aging, playing a significant role in the progression of sarcopenia (Maldonado et al., 2023). It interacts with several key hallmarks of aging in muscle, including mitochondrial dysfunction, loss of proteostasis (the ability to maintain protein quality), and cellular senescence (Maldonado et al., 2023; Kadoguchi et al., 2020; Höhn et al., 2017). At the molecular level, oxidative stress causes a disruption in the equilibrium that exists between the synthesis of proteins and their degradation throughout muscle tissue (Meng and Yu, 2010). This imbalance is often characterized by a blunting of anabolic signaling pathways, which promote muscle protein synthesis, and an increase in catabolic signaling pathways, which lead to muscle protein degradation (Meng and Yu, 2010). ROS can initiate proteolytic pathways in muscle cells, encompassing the ubiquitin-proteasome system and the functions of caspases and calpains, resulting in the degradation of muscle proteins (Gomes et al., 2017; Khalil and Xiao, 2018; Smuder et al., 2010).

Mitochondrial dysfunction, a hallmark of aging, is both a source and a consequence of oxidative stress in muscle (Maldonado et al., 2023). Oxidative damage to mitochondria impairs their ability to produce ATP efficiently, leading to energy deficits within the muscle cells (Hyatt et al., 2019). This dysfunction leads to elevated ROS production, establishing a detrimental cycle in which oxidative stress inflicts damage on mitochondria, subsequently causing increased ROS production and further worsening the issue (Maldonado et al., 2023). The direct damage caused by ROS to various muscle cell components, including DNA, proteins, and lipids, further impairs muscle structure and function (Chen et al., 2022a; Powers et al., 2011).

The capacity of muscle to regenerate and repair itself is also compromised by oxidative stress (Kozakowska et al., 2015). Satellite cells, the stem cells of muscle tissue responsible for regeneration, are negatively affected by ROS, leading to impaired muscle repair and maintenance (Shengchen et al., 2021). Furthermore, inflammation, a common feature of aging, often interacts with and exacerbates oxidative stress in muscle (Maldonado et al., 2023). This interplay can lead to anabolic resistance, further contributing to muscle loss (Chen et al., 2022a). Interestingly, different types of muscle fibers may exhibit varying susceptibility to oxidative stress with age. Type II muscle fibers, which are important for strength and power, tend to decline more rapidly with age, potentially due to a greater vulnerability to oxidative injury compared to Type I fibers (Gomes et al., 2017). Increased ROS production with age (from mitochondria, NADPH oxidase, etc.) leads to oxidative damage of cellular components (DNA, proteins, lipids) and mitochondrial dysfunction (Hajam et al., 2022; Zia et al., 2022). This imbalance between protein synthesis and breakdown (increased catabolism, decreased anabolism), activation of proteolytic pathways (UPS, caspases), impaired muscle regeneration and satellite cell function, and increased inflammation cause muscle fiber atrophy and loss, leading to sarcopenia (Wang et al., 2017).

## 6 The nexus of oxidative stress, frailty, and sarcopenia

Sarcopenia is recognized as a significant factor in frailty among older adults, exhibiting a robust association and overlap between the two conditions (Gomes et al., 2017). The reduction in muscle mass and strength associated with sarcopenia is a fundamental aspect of frailty syndrome, leading to increased weakness, slowness, and heightened risk of falls and disability (Cunha et al., 2023). Sarcopenia is a significant predictor of frailty, as well as poor quality of life and increased mortality in the elderly (Gomes et al., 2017). It appears that frail people have higher oxidative stress levels than healthy people (Anwar et al., 2023). Accordingly, frailty may develop or worsen as a result of an elevated burden of oxidative stress.

Inflammatory pathways, often influenced or intensified by oxidative stress, are correlated with the development of sarcopenia and frailty (Chen et al., 2022a; Antuña et al., 2022). The chronic low-grade inflammation often observed in aging, sometimes referred to as “inflammaging,” can contribute to muscle wasting and overall physiological decline, both of which are hallmarks of frailty (Chen et al., 2022a; Baechle et al., 2023). The “direct-hit” hypothesis proposes that ROS-induced direct damage to muscle tissue may contribute to sarcopenia, particularly in frail older adults (Ershler, 2007). In frail elderly women, elevated protein carbonylation, an indicator of reactive oxygen species-induced muscle damage, was associated with reduced grip strength, a marker of muscle mass (Ershler, 2007). This suggests a direct link between oxidative stress and muscle decline in the context of frailty. Furthermore, oxidative stress and inflammation contribute to frailty syndrome and metabolic syndrome, suggesting a syndemic relationship (Dzięgielewska-Gęsiak and Muc-Wierzgoń, 2023; Dao et al., 2022).

While oxidative stress is a significant factor, other physiological and lifestyle elements also contribute to the interconnectedness of sarcopenia and frailty (Chen et al., 2020; Ward et al., 2022). Factors include decreased physical activity, hormonal alterations, insulin resistance, and nutritional deficiencies (Gomes et al., 2017). Sarcopenia and frailty are linked to sirtuins, cellular regulators (Anwar et al., 2023). These proteins are thought to regulate oxidative stress, inflammation, mitochondrial function, and muscle repair, indicating their potential as therapeutic targets for the prevention or mitigation of sarcopenia and frailty (Anwar et al., 2023). Building on these links, exercise interventions offer a targeted strategy to interrupt this nexus, as explored next.

## 7 Exercise interventions: a strategy to combat oxidative stress, muscle aging, and frailty

Exercise interventions represent a viable approach to address oxidative stress, muscle aging, and frailty among the elderly population (El Assar et al., 2022; Viña et al., 2016). Multiple studies indicate that exercise training effectively reduces oxidative damage and enhances antioxidant capacity in older adults (Militello et al., 2024; Amiri and Sheikholeslami-Vatani, 2023; Ruangthai and Phoemsapthawee, 2019). Aerobic exercise, in particular, has shown effectiveness in reducing oxidant markers and increasing antioxidant markers in older populations (Ye et al., 2021). Resistance training boosts the antioxidant system, which is crucial (Cuyul-Vásquez et al., 2020). The integration of aerobic and resistance training has demonstrated beneficial effects on redox biomarkers, as evidenced by reductions in pro-oxidant markers and increases in antioxidant enzymes (Atashak and Azizbeigi, 2017). Even mind-body exercises like Tai Chi and Baduanjin have shown potential in improving oxidative stress markers in individuals (Lu et al., 2021; Chen et al., 2022b). Current research investigates the effects of increased physical activity on oxidative stress biomarkers among diverse populations of older adults, including those living independently (Campbell et al., 2010). A 12-week physical exercise program in elderly women demonstrated the ability to attenuate oxidative stress by increasing levels of sulfhydryl (SH) groups, a marker of antioxidant capacity (Rodziewicz-Flis et al., 2022).

In terms of muscle health, resistance exercise is considered a first-line treatment for counteracting sarcopenia (Gomes et al., 2017). An integrated approach incorporating aerobic, resistance, balance, and flexibility exercises is often recommended to improve various aspects of physical functioning in every individuals (De et al., 2011). Multicomponent exercise programs that integrate various exercise types have demonstrated effectiveness in enhancing muscle strength, endurance, stability, and mobility among the elderly (Li et al., 2023). Notably, resistance exercise can boost strength in people as old as 90, showing that muscle health interventions can be beneficial at any age (Peterson et al., 2011).

Physical activity has also demonstrated the capacity to help reverse the effects of frailty in older adults (Liu and Fielding, 2011). Exercise regimens with multiple components greatly improve frailty status (Prommaban et al., 2024). Exercise interventions can enhance strength, walking speed, and nutritional status in frail and pre-frail adults (Álvarez-Satta et al., 2020). Specific exercise types, such as Baduanjin, have shown promise in improving cognitive frailty (Ye et al., 2024).

For frail elderly individuals, specific exercise recommendations emphasize multicomponent programs that include aerobic, resistance, balance, and flexibility training (Prommaban et al., 2024). Low-intensity options, such as aquatic exercise, may be particularly suitable for those with mobility limitations (Di Lorito et al., 2021; Heywood et al., 2017). Recommendations for frequency and duration typically correspond with guidelines from organizations such as the ACSM and the WHO, which advocate for a minimum of 150 min of moderate aerobic activity or 75 min of vigorous activity weekly, in addition to resistance training on at least 2 days per week (Di Lorito et al., 2021). Tailored programs and gradual progression are crucial considerations (Yang, 2019). Mind-body exercises like Tai Chi and Baduanjin can also be valuable components of an exercise regimen for frail older adults (Landi et al., 2010) (Figure 2; Tables 1, 2).

## 8 Biomarkers: tracking oxidative stress and muscle health in exercising elderly individuals

Various biomarkers are applicable for monitoring alterations in oxidative stress and muscle health among elderly individuals engaged in exercise programs, with translational potential for clinical screening and intervention monitoring (Calvani et al., 2015; Picca et al., 2022). However, biomarker responses vary by study subjects (e.g., frail vs. healthy elderly show higher baseline MDA), exercise prescriptions (e.g., aerobic reduces circulating MDA more than resistance), and monitoring methods (e.g., plasma vs. muscle biopsy for SOD) (Calvani et al., 2018; Marzetti et al., 2013). These differences lead to ambiguous evidence; for instance, neutral effects in frail cohorts may stem from comorbidities blunting adaptations, while trained individuals exhibit amplified increases (Calvani et al., 2018). Selection criteria include sensitivity (e.g., MDA for acute changes), specificity (e.g., 8-OHdG for DNA damage), non-invasiveness (blood-based preferred), and clinical correlation (e.g., with frailty scores) (Marzetti et al., 2013). Application differs: MDA/SOD for general redox monitoring in community settings, irisin for muscle-specific regeneration in sarcopenia trials (Calvani et al., 2018; Marmondi et al., 2025). Substantive analysis clarifies: e.g., MDA suits low-intensity programs in frail populations due to rapid detectability, while SOD better tracks chronic adaptations in multicomponent interventions (Marzetti et al., 2013).

MDA serves as a widely recognized marker of oxidative stress. Research indicates that its levels decline with regular exercise in older adults (Ye et al., 2021). Other biomarkers of oxidative stress include LPO, 8-OHdG, and 8-isoPGF2 (Ye et al., 2021; Girotti and Korytowski, 2023; Bláhová et al., 2023). Antioxidant enzymes like SOD, GPx, and GR are also important biomarkers, with exercise often leading to an increase in their activity (Xie et al., 2025). Total antioxidant capacity (TAC) and levels of NO and SH groups are additional indicators of the body’s antioxidant status that can be influenced by exercise (Ye et al., 2021). Levels of Vitamin C and E may also be measured, although their response to exercise in the elderly can be variable (Ye et al., 2021). Promising translational biomarkers include MDA and SOD for oxidative stress (correlated with frailty in cohort studies) and irisin for muscle regeneration (elevated post-exercise, linked to sarcopenia reversal) (Marmondi et al., 2025; Liu et al., 2016).

Various biomarkers are applicable for monitoring alterations in oxidative stress and muscle health among elderly individuals engaged in exercise programs. MDA serves as a widely recognized marker of oxidative stress. Research indicates that its levels decline with regular exercise in older adults (Ye et al., 2021). Other biomarkers of oxidative stress include LPO, 8-OHdG, and 8-isoPGF2 (Ye et al., 2021; Girotti and Korytowski, 2023; Bláhová et al., 2023). Antioxidant enzymes like SOD, GPX, and GR are also important biomarkers, with exercise often leading to an increase in their activity (Xie et al., 2025). Total antioxidant capacity (TAC) and levels of NO and SH groups are additional indicators of the body’s antioxidant status that can be influenced by exercise (Ye et al., 2021). Levels of Vitamin C and E may also be measured, although their response to exercise in the elderly can be variable (Ye et al., 2021).

Biomarkers of muscle aging and health that can be influenced by exercise include CK and LDH, which are often used as markers of muscle injury (Ni et al., 2023; Bouzid et al., 2014b). MCSA and MQ, evaluated via imaging techniques, can indicate alterations in muscle mass and composition due to exercise (Carru et al., 2018). Measures of muscle function, such as MVC and MIT, can also be used to track the benefits of exercise interventions (Carru et al., 2018; Liu et al., 2023). Changes in lean body mass and muscle fiber size are direct indicators of muscle health and can be assessed through various methods (Gomes et al., 2017). Myokines, including Irisin, are released by muscle contraction during exercise and are emerging as potential biomarkers indicative of the beneficial effects of physical activity on muscle and overall health (El Assar et al., 2022).

Exercise interventions have been shown to lower MDA and increase SOD (Ye et al., 2021; Thirupathi et al., 2021; Sekineh et al., 2013). In older adults, baseline MDA, homocysteine, and taurine levels have been linked to exercise-induced muscle mass and function changes (Carru et al., 2018). Combined training reduces oxidative stress and inflammatory markers, demonstrating its systemic benefits (Cheragh Birjandi et al., 2023). Exercise can also affect skeletal muscle autophagy markers, which are essential for muscle health (Table 3) (Angulo et al., 2020).

**TABLE 3:** Oxidative stress and muscle aging biomarkers and exercise response in the elderly.

Biomarker type	Specific biomarker	Response to regular exercise	Ref
Oxidative stress	Malondialdehyde (MDA)	Decrease	Ye et al. (2021)
Oxidative stress	Lipid peroxide (LPO)	Decrease	Ye et al. (2021)
Oxidative stress	8-hydroxy-2′-deoxyguanosine (8-OHdG)	Decrease	Ye et al. (2021)
Oxidative stress	8-iso-prostaglandin F2α (8-isoPGF2)	Decrease	Ye et al. (2021)
Oxidative stress	Superoxide dismutase (SOD)	Increase	Ye et al. (2021)
Oxidative stress	Glutathione peroxidase (GPX)	Increase	Bouzid et al. (2014b)
Oxidative stress	Glutathione reductase (GR)	Increase	Bouzid et al. (2014b)
Oxidative stress	Total antioxidant capacity (TAC)	Increase	Ye et al. (2021)
Oxidative stress	Nitric oxide (NO)	Increase	Ye et al. (2021)
Oxidative stress	Sulfhydryl (SH) groups	Increase	Rodziewicz-Flis et al. (2022)
Muscle injury	Creatine kinase (CK)	Mixed/Increase	Ni et al. (2023)
Muscle injury	Lactate dehydrogenase (LDH)	Mixed	Ni et al. (2023)
Muscle mass/function	Muscle cross-sectional area (MCSA)	Increase	Carru et al. (2018)
Muscle mass/function	Muscle quality (MQ)	Increase	Carru et al. (2018)
Muscle mass/function	Maximal voluntary contraction (MVC)	Increase	Carru et al. (2018)
Muscle mass/function	Isokinetic torque (MIT)	Increase	Carru et al. (2018)
Muscle mass/function	Lean body mass	Increase	Gomes et al. (2017)
Muscle mass/function	Muscle fiber size	Increase	De et al. (2011)
Muscle health	Irisin	Increase	Ning et al. (2022)
Muscle health	Autophagy markers	Increase	Angulo et al. (2020)

## 9 Discussion: synthesis of current evidence and future research directions

### 9.1 Summary of key findings

The current body of scientific literature, including numerous review articles and meta-analyses, generally supports the notion that regular exercise has a positive effect on reducing oxidative stress and improving muscle health in the elderly (Militello et al., 2024; Simioni et al., 2018; Morucci et al., 2022). Consistent findings highlight that physical activity can enhance antioxidant defense mechanisms and reduce basal levels of oxidative stress in older adults compared to sedentary individuals (Militello et al., 2024). Various forms of exercise, such as aerobic, resistance, combined training, and mind-body practices like Tai Chi and Baduanjin, have shown the ability to influence oxidative stress markers and enhance muscle health (Ye et al., 2021). However, the literature also presents some contradictions and inconsistencies regarding the optimal type, frequency, and intensity of exercise, as well as the specific effects on various biomarkers (Ye et al., 2021).

### 9.2 In-depth analysis of mechanisms

Exercise modulates redox homeostasis through Nrf2/AMPK/PGC-1α pathways, reducing mitochondrial ROS and inflammation while promoting biogenesis (Militello et al., 2024; Zhang et al., 2024; Huang et al., 2019; Neto et al., 2023; Halling et al., 2019; El Assar et al., 2022; Ya et al., 2022; Kitaoka, 2021). This counters sarcopenia by balancing protein turnover and frailty by enhancing reserves (Anwar et al., 2023; Gomes et al., 2017). However, mechanisms vary by exercise type; e.g., resistance emphasizes proteostasis, aerobic targets mitochondria.

### 9.3 Conflicting or neutral findings

Despite overall benefits, the literature presents some contradictions and inconsistencies regarding the optimal type, frequency, and intensity of exercise, as well as the specific effects on various biomarkers (Ye et al., 2021). For instance, while meta-analyses show aerobic exercise reduces MDA, some RCTs report neutral effects on running-specific markers, possibly due to intensity thresholds or individual heterogeneity (e.g., baseline fitness, genetics) (Ye et al., 2021; Spirlandeli et al., 2014). Chronic exercise from middle to old age may initially increase oxidative damage in untrained individuals but yields net benefits long-term; neutral findings in frail subgroups highlight need for tailored interventions (Bláhová et al., 2023). Heterogeneous responses underscore that not all older adults benefit equally, influenced by comorbidities or training status (Wang et al., 2021b; Thirupathi et al., 2021).

### 9.4 Translational implications

Translational perspectives include using biomarkers like MDA/SOD for frailty screening and monitoring exercise response, with pathways like PGC-1α offering targets for adjunct therapies (e.g., Nrf2 activators) (Calvani et al., 2015; Picca et al., 2022; Calvani et al., 2018; Marmondi et al., 2025; Liu et al., 2016). Clinically, this supports integrating exercise into geriatric care to delay sarcopenia/frailty progression, with potential for personalized programs based on biomarker profiles.

### 9.5 Gaps and future directions

Several gaps remain in our current understanding. There is a need for more research to definitively establish the best frequency, type, and intensity of exercise for preventing or treating age-induced skeletal muscle alterations and frailty (Gomes et al., 2017). Further verification is required to ascertain the specific effects of different forms of aerobic exercise on oxidative stress in older adults (Ye et al., 2021). The optimal exercise program variables for frail older adults remain unclear (Prommaban et al., 2024). Additionally, little is known about the long-term effects of exercise programs and how they affect cognitive function and inflammatory pathways in this vulnerable population (Prommaban et al., 2024). Current literature on sirtuins and sarcopenia and frailty is contradictory and needs further study (Anwar et al., 2023).

Longitudinal studies should examine how exercise interventions affect oxidative stress, muscle health, and frailty in the elderly (Simioni et al., 2018). Examining the molecular mechanisms by which exercise influences oxidative stress and its effects on muscle aging and frailty is essential for creating targeted and effective interventions (Simioni et al., 2018). Future studies should also explore personalized exercise prescriptions based on individual characteristics, health status, and biomarker responses. Examining the impact of novel exercise modalities and further investigating the role of potential biomarkers for monitoring exercise effectiveness and predicting individual responses are also important avenues for future research.

## 10 Limitations

This narrative review has several limitations. The non-systematic approach may introduce selection bias, as literature was chosen qualitatively without formal meta-analysis or risk of bias assessment (e.g., PRISMA). Reliance on heterogeneous studies (varying designs, populations) limits generalizability, and conflicting findings were not fully resolved. Lack of primary data means conclusions are interpretive. Long-term exercise effects and underrepresented groups (e.g., very frail or non-Western populations) are underexplored. While evidence strength is generally moderate-high for key interventions (e.g., aerobic/combined), the narrative format limits quantitative rigor; however, the expanded methods enhance reference value. Future systematic reviews could address these.

## 11 Conclusion

In conclusion, the evidence reviewed in this report underscores the complex yet generally positive role of exercise in modulating oxidative stress and its impact on muscle aging and frailty in elderly individuals. Regular physical activity, including aerobic, resistance, and mind-body exercises, enhances antioxidant defenses, reduces oxidative damage, improves muscle health, and may mitigate frailty. Exercise appears to counteract the detrimental effects of aging on muscle by influencing key mechanisms, including protein turnover, mitochondrial function, and inflammation, often linked to oxidative stress. Sarcopenia, a major contributor to frailty, can be effectively addressed through exercise interventions, particularly resistance training.

The necessity of this study stems from the growing aging population and rising sarcopenia/frailty burden, necessitating evidence-based strategies. Practical objectives include guiding tailored exercise prescriptions (e.g., multicomponent programs) for clinical and public health use. Innovations lie in integrating redox mechanisms (Nrf2/AMPK/PGC-1α) with translational biomarkers, contributing a comprehensive synthesis for future interventions to enhance independence and wellbeing.

The significance of physical activity in promoting healthy aging is paramount. Regular exercise is fundamental to strategies aimed at reducing oxidative stress, enhancing muscle health, and preventing frailty in older adults. These insights carry important clinical and public health implications, guiding the formulation of effective exercise programs and public health recommendations to improve independence and quality of life for the elderly. Despite considerable advancements in understanding this intricate relationship, ongoing research remains essential to refine knowledge, address gaps, and create personalized exercise strategies for optimal outcomes in older individuals. Continued investigation into molecular mechanisms and the identification of reliable biomarkers will be crucial for advancing this field and enhancing the health and wellbeing of the aging population.
