# Supplementary material for: Redox regulation in aging muscles: exercise as a key modulator to combat sarcopenia and frailty
Source: Front Cell Dev Biol. 2026 Mar 17;14:1772623. doi: 10.3389/fcell.2026.1772623 (PMC13036230; doi:10.3389/fcell.2026.1772623)
Supplement: Supplementary file 1 [file Table1.docx]

Supplementary Table S1: Details of the 104 included articles in the narrative review. This table provides an overview of the selected studies, including authors, publication year, study design, target population, and key findings relevant to oxidative stress, exercise interventions, sarcopenia, and frailty in older adults (≥60 years). The articles were synthesized thematically to support the review's objectives, with a focus on high-quality evidence from RCTs, meta-analyses, and reviews. Full references are available in the main manuscript's reference list where cited.

| **Ref No** | **Authors** | **Year** | **Study Design** | **Population** | **Key Findings** |
| --- | --- | --- | --- | --- | --- |
| 1 | Militello R, Luti S, Gamberi T, Pellegrino A, Modesti A, Modesti PA | 2024 | Review | Older adults | Physical activity reduces oxidative stress in aging through enhanced antioxidant defenses. |
| 2 | Simioni C, Zauli G, Martelli AM, Vitale M, Sacchetti G, Gonelli A, et al. | 2018 | Review | Adults and older adults | Exercise and antioxidants mitigate oxidative stress in aging. |
| 3 | Maldonado E, Morales-Pison S, Urbina F, Solari A | 2023 | Review | Older adults | Oxidative stress plays a key role in aging hallmarks, including sarcopenia. |
| 4 | Anwar M, Pradhan R, Dey S, Kumar R | 2023 | Review | Older adults with sarcopenia/frailty | Sirtuins regulate oxidative stress and frailty. |
| 5 | Álvarez-Satta M, Berna-Erro A, Carrasco-Garcia E, Alberro A, Saenz-Antoñanzas A, Vergara I, et al. | 2020 | Review | Frail older adults | Oxidative stress and inflammation contribute to frailty. |
| 6 | Dai X, Hu Y, Jiang L, Lei L, Fu C, Wu S, et al. | 2023 | Observational | Older adults | Decreased oxidative stress response in aging skin. |
| 7 | Ye Y, Lin H, Wan M, Qiu P, Xia R, He J, et al. | 2021 | Meta-analysis | Older adults | Aerobic exercise reduces oxidative stress markers. |
| 8 | Gomes MJ, Martinez PF, Pagan LU, Damatto RL, Cezar MDM, Lima ARR, et al. | 2017 | Review | Older adults with sarcopenia | Oxidative stress influences skeletal muscle aging; exercise mitigates it. |
| 9 | Chen LK, Woo J, Assantachai P, Auyeung TW, Chou MY, Iijima K, et al. | 2020 | Consensus | Older adults | Updated sarcopenia diagnosis and treatment guidelines. |
| 10 | Kadoguchi T, Shimada K, Miyazaki T, Kitamura K, Kunimoto M, Aikawa T, et al. | 2020 | Observational | Aging mice (translational to older adults) | Oxidative stress linked to mitochondrial dysfunction and muscle atrophy. |
| 11 | Maldonado E, Morales-Pison S, Urbina F, Solari A | 2023 | Review | Older adults | Aging hallmarks and oxidative stress role. |
| 12 | Shengchen W, Jing L, Yujie Y, Yue W, Shiwen X | 2021 | Experimental | Muscle cells (translational to older adults) | ROS overproduction disrupts muscle regeneration. |
| 13 | Ji LL | 2001 | Review | Older adults | Exercise alleviates oxidative stress in old age. |
| 14 | Atashak S, Azizbeigi K | 2017 | RCT | Elderly men | Concurrent exercise reduces oxidative stress biomarkers. |
| 15 | Ye Y, Lin H, Wan M, Qiu P, Xia R, He J, et al. | 2021 | Meta-analysis | Older adults | Aerobic exercise effects on oxidative stress. |
| 16 | Álvarez-Satta M, Berna-Erro A, Carrasco-Garcia E, Alberro A, Saenz-Antoñanzas A, Vergara I, et al. | 2020 | Review | Frail older adults | Oxidative stress in frailty models. |
| 17 | Prommaban A, Moonkayaow S, Phinyo P, Siviroj P, Sirikul W, Lerttrakarnnon P | 2024 | Systematic review | Older adults | Exercise interventions on frailty and biomarkers. |
| 18 | Ji LL | 2001 | Review | Older adults | Exercise impact on oxidative stress. |
| 19 | Zhang Y, Zhang H, Zhao F, Jiang Z, Cui Y, Ou M, et al. | 2023 | Experimental | Models (translational) | Mitochondrial-targeted therapy for oxidative stress. |
| 20 | Chistiakov DA, Sobenin IA, Revin VV, Orekhov AN, Bobryshev YV | 2014 | Review | Older adults | Mitochondrial aging and dysfunction. |
| 21 | Kozakiewicz M, Maciej K, Olga K, Kędziora-Kornatowska K | 2019 | Observational | Advanced age people | Changes in blood antioxidant defense. |
| 22 | Sullivan-Gunn MJ, Lewandowski PA | 2013 | Observational | Aging models | Decreased antioxidants in sarcopenia. |
| 23 | Mao C, Yuan J-Q, Lv Y-B, Gao X, Yin Z-X, Kraus VB, et al. | 2019 | Cohort | Older adults | SOD, MDA associated with mortality. |
| 24 | Shin MH, Moon YJ, Seo J-E, Lee Y, Kim KH, Chung JH | 2008 | Experimental | Cells | ROS from NADPH/xanthine oxidase in stress. |
| 25 | Vida C, Rodríguez-Terés S, Heras V, Corpas I, De la Fuente M, González E | 2011 | Observational | Aging mice | Xanthine oxidase increase in aging. |
| 26 | Chen M, Wang Y, Deng S, Lian Z, Yu K | 2022 | Review | Older adults | Oxidative stress and inflammation in muscle aging. |
| 27 | Ni C, Ji Y, Hu K, Xing K, Xu Y, Gao Y | 2023 | Systematic review/meta-analysis | Elderly | Exercise and antioxidants on lipid peroxidation. |
| 28 | Ammendolia DA, Bement WM, Brumell JH | 2021 | Review | General | Plasma membrane integrity in disease. |
| 29 | Ershler WB | 2007 | Review | Older adults | Oxidative stress and inflammation in frailty. |
| 30 | Davies MJ | 2016 | Review | General | Protein oxidation. |
| 31 | Benkafadar N, François F, Affortit C, Casas F, Ceccato JC, Menardo J, et al. | 2019 | Experimental | Models | ROS-induced DNA damage in senescence. |
| 32 | Junior MDF, Cavalcante KVN, Ferreira LA, Lopes PR, Pontes CNR, Bessa ASM, et al. | 2019 | Experimental | Rats | Oxidative stress in cardiovascular dysfunction. |
| 33 | Hassan W, Noreen H, Rehman S, Kamal MA, da Rocha JBT | 2022 | Review | General | Oxidative stress in neurological disorders. |
| 34 | Huang Z, Zhou L, Duan J, Qin S, Jiang J, Chen H, et al. | 2024 | Experimental | Models | Oxidative stress in cancer metastasis. |
| 35 | Soysal P, Isik AT, Carvalho AF, Fernandes BS, Solmi M, Schofield P, et al. | 2017 | Systematic review | Frail older adults | Oxidative stress and frailty. |
| 36 | Bouzid MA, Filaire E, McCall A, Fabre C | 2015 | Review | Older adults | ROS in exercise and aging. |
| 37 | Powers SK, Jackson MJ | 2008 | Review | General | Exercise-induced oxidative stress. |
| 38 | Henríquez-Olguin C, Knudsen JR, Raun SH, Li Z, Dalbram E, Treebak JT, et al. | 2019 | Experimental | Models | NADPH oxidase in muscle glucose uptake. |
| 39 | Gomez-Cabrera MC, Close GL, Kayani A, McArdle A, Viña J, Jackson MJ | 2010 | Experimental | Models | Xanthine oxidase in muscle force. |
| 40 | Spirlandeli AL, Deminice R, Jordao AA | 2014 | Observational | Adults | MDA as biomarker post-exercise. |
| 41 | Xu Y, Liang M, Ugbolue UC, Fekete G, Gu Y | 2022 | Systematic review/meta-analysis | Healthy adults | Exercise intensity on SOD. |
| 42 | Shields N, Downs J, de Haan JB, Taylor NF, Torr J, Fernhall B, et al. | 2018 | Systematic review/meta-analysis | Down syndrome (older) | Exercise on oxidative stress. |
| 43 | Wibawa J, Febrianto N, Fudin M, Ockta Y, Festiawan R | 2024 | Systematic review | General | Exercise increases glutathione peroxidase. |
| 44 | Grossini E, Venkatesan S, Pour MMO, Conti A, Concina D, Opizzi A, et al. | 2024 | RCT | Older adults in long-term care | Lifestyle intervention on redox balance. |
| 45 | Wang F, Wang X, Liu Y, Zhang Z | 2021 | Review | General | Exercise-induced ROS on muscle functions. |
| 46 | Wang X, Wang Z, Tang D | 2021 | Experimental | Mice with COPD (translational) | Aerobic exercise alleviates oxidative stress. |
| 47 | Lima JE, Moreira NC, Sakamoto-Hojo ET | 2022 | Review | General | Mechanisms in type 2 diabetes including oxidative stress. |
| 48 | Thirupathi A, Wang M, Lin JK, Fekete G, István B, Baker JS, et al. | 2021 | Systematic review | General | Exercise modalities on oxidative stress. |
| 49 | El Assar M, Álvarez-Bustos A, Sosa P, Angulo J, Rodríguez-Mañas L | 2022 | Review | Older adults | Physical activity on oxidative stress in muscle aging. |
| 50 | Lu Z, Xu Y, Song Y, Bíró I, Gu Y | 2021 | Meta-analysis | Patients with oxidative stress diseases | Exercise intensities on oxidative stress. |
| 51 | Rosado-Pérez J, Castelán-Martínez OD, Mújica-Calderón AJ, Sánchez-Rodríguez MA, Mendoza-Núñez VM | 2021 | Systematic review/meta-analysis | General | Tai Chi on oxidative stress markers. |
| 52 | Kitaoka Y | 2021 | Review | General | Nrf2 in skeletal muscle exercise capacity. |
| 53 | Huang DD, Fan SD, Chen XY, Yan XL, Zhang XZ, Ma BW, et al. | 2019 | Experimental | Aging models | Nrf2 deficiency exacerbates frailty and sarcopenia. |
| 54 | El Assar M, Álvarez-Bustos A, Sosa P, Angulo J, Rodríguez-Mañas L | 2022 | Review | Older adults | Exercise on oxidative stress and inflammation. |
| 55 | Neto IVS, Pinto AP, Muñoz VR, de Cássia Marqueti R, Pauli JR, Ropelle ER, et al. | 2023 | Review | General | PGC-1α actions in exercise and aging. |
| 56 | Halling JF, Jessen H, Nøhr-Meldgaard J, Buch BT, Christensen NM, Gudiksen A, et al. | 2019 | Experimental | Older adults | PGC-1α in mitochondrial properties with aging/exercise. |
| 57 | Gureev AP, Shaforostova EA, Popov VN | 2019 | Review | General | Nrf2 and PGC-1α in mitochondrial biogenesis. |
| 58 | Bouzid MA, Hammouda O, Matran R, Robin S, Fabre C | 2014 | Observational | Older adults | Low-intensity aerobic exercise on oxidative stress. |
| 59 | Zhao M, Xiao M, Tan Q, Lyu J, Lu F | 2023 | Systematic review/meta-analysis | CKD patients (older) | Aerobic exercise on oxidative stress in CKD. |
| 60 | Baghaiee B, Botelho Teixeira AM, Tartibian B | 2016 | RCT | Middle-aged men | Moderate aerobic exercise increases SOD, decreases MDA. |
| 61 | Ghoraba R, Karami-Mohajeri S, Behdarvand A, Saber A | 2022 | Review | Hemodialysis patients (older) | Aerobic exercise on oxidative stress in hemodialysis. |
| 62 | Ozkol MZ, Turgay F, Varol SR, Ozcaldiran B, Vural F, Aksit T, et al. | 2012 | Observational | Adults | Aerobic/anaerobic exercise on nitric oxide. |
| 63 | Vezzoli A, Mrakic-Sposta S, Montorsi M, Porcelli S, Vago P, Cereda F, et al. | 2019 | RCT | Older individuals | Resistive training reduces oxidative stress, improves muscle. |
| 64 | Di Lorito C, Long A, Byrne A, Harwood RH, Gladman JRF, Schneider S, et al. | 2021 | Systematic review | Older adults | Exercise interventions for older adults. |
| 65 | Atashak S, Azizbeigi K | 2017 | RCT | Elderly men | Concurrent training on oxidative stress. |
| 66 | Bachi ALL, Barros MP, Vieira RP, Rocha GA, de Andrade PBM, Victorino AB, et al. | 2019 | RCT | Elderly women | Combined training reduces redox and cytokines. |
| 67 | Cheragh Birjandi K, Ghasemi E, Karimi E, Ghoncheh S | 2023 | RCT | Elderly men | Combined training reduces biomarkers. |
| 68 | Lu Z, Xu Y, Song Y, Bíró I, Gu Y | 2021 | Systematic review | Patients with oxidative stress | Exercise types on oxidative stress. |
| 69 | Rosado-Pérez J, Castelán-Martínez OD, Mújica-Calderón AJ, Sánchez-Rodríguez MA, Mendoza-Núñez VM | 2021 | Systematic review/meta-analysis | General | Tai Chi on oxidative stress. |
| 70 | Gupta R, Maurya PK | 2020 | Review | General | Yoga on oxidative stress in aging. |
| 71 | Ye Y, Wan M, Lin H, Xia R, He J, Qiu P, et al. | 2024 | RCT | Older adults with cognitive frailty | Baduanjin on oxidative stress and frailty. |
| 72 | Höhn A, Weber D, Jung T, Ott C, Hugo M, Kochlik B, et al. | 2017 | Review | General | Oxidative stress, proteostasis, senescence in aging. |
| 73 | Meng SJ, Yu LJ | 2010 | Review | General | Oxidative stress and inflammation in sarcopenia. |
| 74 | Khalil R | 2018 | Review | General | Ubiquitin-proteasome in muscle atrophy. |
| 75 | Smuder AJ, Kavazis AN, Hudson MB, Nelson WB, Powers SK | 2010 | Experimental | Models | Oxidation in protein degradation. |
| 76 | Hyatt H, Deminice R, Yoshihara T, Powers SK | 2019 | Review | General | Mitochondrial dysfunction in inactivity. |
| 77 | Powers SK, Ji LL, Kavazis AN, Jackson MJ | 2011 | Review | General | ROS impact on skeletal muscle. |
| 78 | Kozakowska M, Pietraszek-Gremplewicz K, Jozkowicz A, Dulak J | 2015 | Review | General | Oxidative stress in muscle injury/regeneration. |
| 79 | Chen M, Wang Y, Deng S, Lian Z, Yu K | 2022 | Review | Older adults | Antioxidant therapy in muscle aging. |
| 80 | Hajam YA, Rani R, Ganie SY, Sheikh TA, Javaid D, Qadri SS, et al. | 2022 | Review | General | Oxidative stress in pathology and aging. |
| 81 | Zia A, Farkhondeh T, Pourbagher-Shahri AM, Samarghandian S | 2022 | Review | General | Mitochondrial dysfunction in aging. |
| 82 | Wang J, Leung K-S, Chow SK-H, Cheung W-H | 2017 | Review | Older adults | Inflammation in sarcopenia. |
| 83 | Gomes MJ, Martinez PF, Pagan LU, Damatto RL, Mariano Cezar MDD, Ruiz Lima AR, et al. | 2017 | Review | Older adults | Oxidative stress and exercise in muscle aging. |
| 84 | Cunha AND, Zanetti ML, Santos JLF, Rodrigues RAP | 2023 | Epidemiological | Older adults with/without diabetes | Frailty and sarcopenia in diabetes. |
| 85 | Antuña E, Cachán-Vega C, Bermejo-Millo JC, Potes Y, Caballero B, Vega-Naredo I, et al. | 2022 | Review | Older adults | Inflammaging in sarcopenia. |
| 86 | Baechle JJ, Chen N, Makhijani P, Winer S, Furman D, Winer DA | 2023 | Review | General | Chronic inflammation in aging. |
| 87 | Dzięgielewska-Gęsiak S, Muc-Wierzgoń M | 2023 | Review | Frail older adults | Inflammation and oxidative stress in frailty/metabolic syndrome. |
| 88 | Dao HHH, Burns MJ, Kha R, Chow CK, Nguyen TN | 2022 | Systematic review/meta-analysis | Older people | Metabolic syndrome and frailty relationship. |
| 89 | Ward DD, Ranson JM, Wallace LMK, Llewellyn DJ, Rockwood K | 2022 | Observational | Older adults | Frailty, lifestyle, genetics in dementia risk. |
| 90 | Viña J, Salvador-Pascual A, Tarazona-Santabalbina FJ, Rodriguez-Mañas L, Gomez-Cabrera MC | 2016 | Review | Older adults | Exercise as drug for frailty. |
| 91 | Amiri E, Sheikholeslami-Vatani D | 2023 | RCT | Older adults | Resistance training and creatine on oxidative stress. |
| 92 | Ruangthai R, Phoemsapthawee J | 2019 | RCT | Elderly with hypertension | Combined exercise on blood pressure and antioxidants. |
| 93 | Cuyul-Vásquez I, Berríos-Contreras L, Soto-Fuentes S, Hunter-Echeverría K, Marzuca-Nassr GN | 2020 | Systematic review/meta-analysis | Older adults | Resistance exercise on redox homeostasis. |
| 94 | Chen D, Zhao G, Fu J, Sun S, Huang X, Su L, et al. | 2022 | Network meta-analysis | Middle-aged/older adults | Traditional Chinese exercise on oxidative stress. |
| 95 | Campbell PT, Gross MD, Potter JD, Schmitz KH, Duggan C, McTiernan A, et al. | 2010 | RCT | Older adults | Exercise on oxidative stress biomarkers. |
| 96 | Rodziewicz-Flis EA, Kawa M, Flis DJ, Szaro-Truchan M, Skrobot WR, Kaczor JJ | 2022 | RCT | Elderly women | Physical exercise attenuates oxidative stress. |
| 97 | de D. Beas-Jiménez J, López-Lluch G, Sánchez-Martínez I, Muro-Jiménez A, Rodríguez-Bies E, Navas P | 2011 | Review | Older adults | Sarcopenia and exercise implications. |
| 98 | Li Y, Gao Y, Hu S, Chen H, Zhang M, Yang Y, et al. | 2023 | Meta-analysis | Frail older adults | Multicomponent exercise on muscle strength/balance. |
| 99 | Peterson MD, Sen A, Gordon PM | 2011 | Meta-analysis | Aging adults | Resistance exercise on lean body mass. |
| 100 | Liu CK, Fielding RA | 2011 | Review | Frail older adults | Exercise as intervention for frailty. |
| 101 | Prommaban A, Moonkayaow S, Phinyo P, Siviroj P, Sirikul W, Lerttrakarnnon P | 2024 | Systematic review | Older adults | Exercise on frailty, outcomes, biomarkers. |
| 102 | Heywood S, McClelland J, Mentiplay B, Geigle P, Rahmann A, Clark R | 2017 | Systematic review/meta-analysis | Adults with musculoskeletal conditions (older) | Aquatic exercise on lower limb strength. |
| 103 | Yang YJ | 2019 | Review | General | Physical activity recommendations in primary care. |
| 104 | Landi F, Abbatecola AM, Provinciali M, Corsonello A, Bustacchini S, Manigrasso L, et al. | 2010 | Review | Frail older adults | Physical activity against frailty. |
