# Supplementary figures and images for: Redox regulation in aging muscles: exercise as a key modulator to combat sarcopenia and frailty
Source: Front Cell Dev Biol. 2026 Mar 17;14:1772623. doi: 10.3389/fcell.2026.1772623 (PMC13036230; doi:10.3389/fcell.2026.1772623)

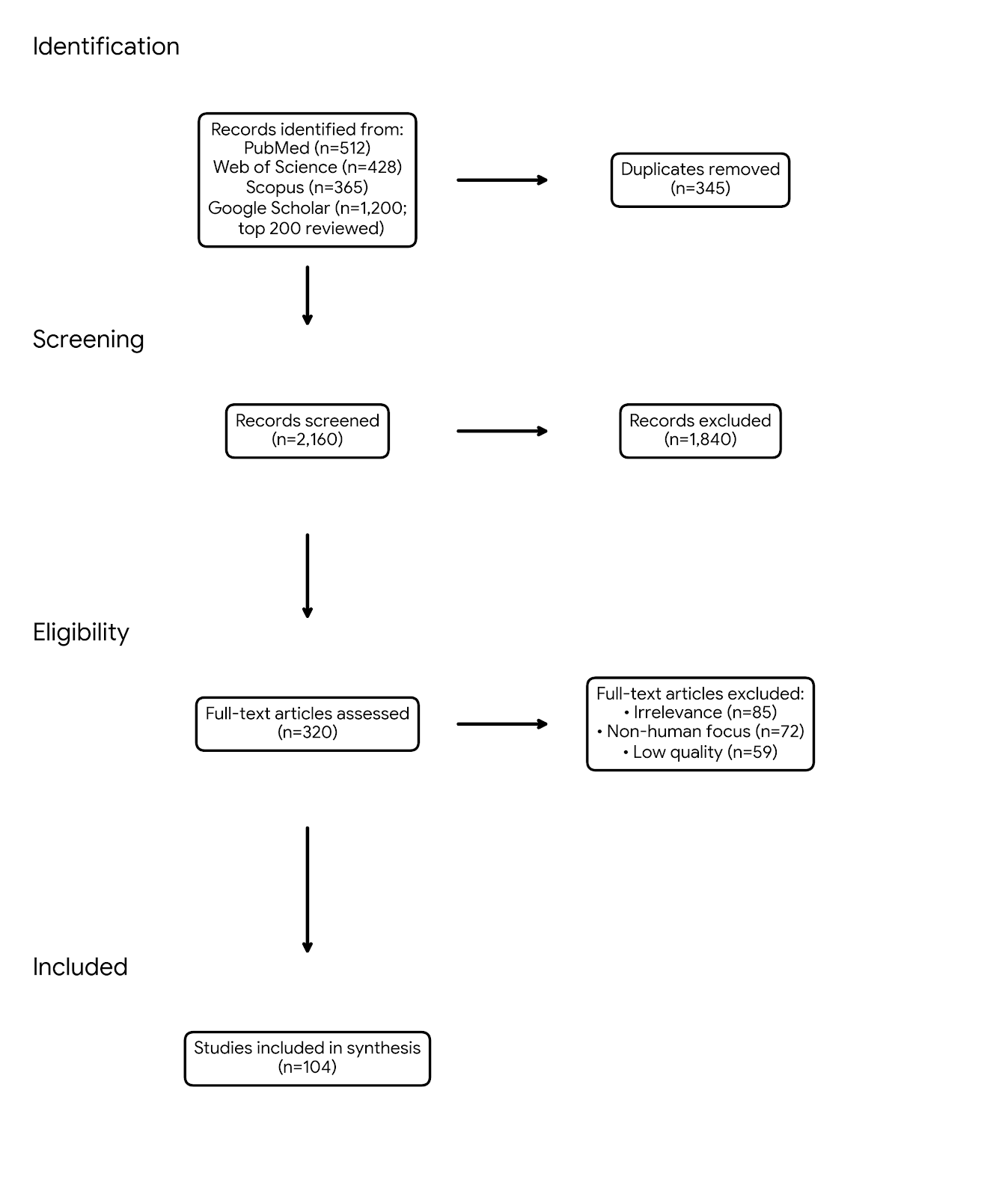

Supplement: Supplementary file 2 [file Image1.tif]
